# Decoding Inflammation: The Role of Neutrophil-to-Lymphocyte Ratio and Platelet-to-Lymphocyte Ratio in Predicting Critical Outcomes in COVID-19 Patients

**DOI:** 10.3390/medicina61040634

**Published:** 2025-03-30

**Authors:** Aida-Isabela Adamescu, Cătălin Tilișcan, Laurențiu Mihăiță Stratan, Nicoleta Mihai, Oana-Alexandra Ganea, Sebastian Ciobanu, Adrian Gabriel Marinescu, Victoria Aramă, Ștefan Sorin Aramă

**Affiliations:** 1 Department II, Pathophysiology and Immunology, Faculty of Dental Medicine, Carol Davila University of Medicine and Pharmacy, 020021 Bucharest, Romania; aida-isabela.adamescu@drd.umfcd.ro (A.-I.A.);; 2 Prof. Dr. Matei Bals National Institute of Infectious Diseases, 021105 Bucharest, Romania; 3 Department II, Infectious Diseases, Faculty of Medicine, Carol Davila University of Medicine and Pharmacy, 020021 Bucharest, Romania; 4 Emergency University Hospital, 050098 Bucharest, Romania

**Keywords:** COVID-19, NLR, PLR, SARS-CoV-2, biomarkers

## Abstract

*Background and Objectives:* The neutrophil-to-lymphocyte ratio (NLR) and platelet-to-lymphocyte ratio (PLR) are novel biomarkers that provide insight into systemic inflammation and how the immune system responds to stress or infection. These ratios have been associated with predicting clinical outcomes in various diseases, including COVID-19. This study aims to evaluate the prognostic value of NLR and PLR in anticipating ICU admission, acute respiratory failure, and disease severity in COVID-19 patients. *Materials and Methods:* We conducted a retrospective, observational study that included 536 patients diagnosed with COVID-19. We analyzed the NLR and PLR values at admission and correlated them with ICU admission, the onset of acute respiratory failure, and clinical outcomes. *Results:* Statistical correlations were identified between elevated NLR and PLR values and the development of complications during hospitalization (*p* = 0.04 and *p* = 0.00), acute hypoxemic respiratory failure (*p* = 0.00), and admission to the intensive care unit (ICU) (*p* = 0.04). No correlations were found between the values of these ratios and mortality (*p* = 0.46 and *p* = 0.32) nor with the development of hepatic cytolysis (*p* = 0.79 and *p* = 0.87). *Conclusions:* NLR and PLR are reliable, easily obtainable biomarkers that can aid in the early prediction of ICU admission and disease severity in COVID-19 patients, offering valuable insights for risk stratification and clinical management. Further prospective studies are needed to validate these biomarkers as part of a broader predictive model for critical care in COVID-19.

## 1. Introduction

The COVID-19 pandemic triggered a unique global response and its public health, social, and economic effects may persist for years [1]. The pandemic also exposed weaknesses in global health infrastructure, highlighting the need for increased awareness for imminent future outbreaks [2]. While we have managed to address many of the immediate effects, the virus continues to evolve quickly, and new variants keep presenting ongoing risks to public health [3].

The immune system plays a key role in fighting against the virus. Initially, the innate immune response, which includes immune cells like neutrophils and macrophages, works to fight the infection. Then, as the disease evolves, the adaptive immune system becomes active, and the T and B lymphocytes specifically target the virus. The problem begins when the immune system is unbalanced, triggering a cytokine storm [4].

In COVID-19, neutrophil levels increase with disease severity, indicating an extraordinary immune system activation due to neutrophil migration to the infection site that is triggered by the intense release of cytokines, while lymphocyte counts decrease in more severe cases [5].

Moreover, very important players of the immune system are thrombocytes, which are not considered a direct part of the immune system but still play significant roles in it. The cytokine storm increases the expression of tissue factor on thrombocytes and releases “procoagulant microvesicles”. Additionally, the increased levels of von Willebrand factor and platelet clumping contribute to intravascular clotting, leading to the formation of “immunothrombi” consisting of platelets and leucocytes [6]. On the other hand, this state of hypercoagulability is also triggered by inflammation and endothelial dysfunction, resulting in conditions like deep vein thrombosis, pulmonary embolism, or even stroke [7]. Studies have shown that, in severe cases, there is a decrease in the platelet count due to the virus’s impact on bone marrow function, inflammation, or as a side effect of certain medications, thereby increasing the risk of hemorrhages [8]. Because of their important roles, neutrophils, lymphocytes, and platelets have been in the spotlight during the pandemic, and numerous studies have been conducted to investigate the predictive value of the NLR and PLR, as they are easy to calculate and offer additional insights into how the disease will progress.

These ratios have been studied not only in the domain of infectious diseases but also in cardiovascular, obstetric, rheumatologic, and oncological settings. A particularly interesting study demonstrated that the pre-interventional assessment of NLR could serve as a predictive biomarker for restenosis in patients with carotid artery stenosis who required revascularization [9]. In the field of rheumatology, elevated PLR has been associated with the presence and severity of rheumatoid arthritis and systemic lupus erythematosus [10]. Another study concluded that higher values of both ratios correlate with greater severity in peripheral artery disease of the lower limbs [11]. Interestingly, high levels of both PLR and NLR were linked to lower chances of remission in type 2 diabetes mellitus following metabolic surgery [12].

Both NLR and PLR provide valuable insights into the inflammatory and immune responses during COVID-19, assisting clinicians in assessing disease severity and potentially predicting outcomes. A study from Turkey found that an NLR value greater than 2.4 is associated with a 20.5-fold higher risk of having COVID-19 [13]. These ratios are easy to measure through routine blood tests and can be used as biomarkers to guide treatment decisions [14,15,16].

Our main objective was to investigate the associations between these ratios and disease severity, the need for hospitalization or ICU admission, and whether they can predict disease progression, complications, or recovery time in COVID-19 patients. Additionally, we explored how combining NLR and PLR might provide a more comprehensive risk assessment, as we found insufficient data in the existing literature on this combined approach.

The retrospective nature of this study presents several opportunities for future prospective research. The findings could serve as a foundation for designing prospective cohort studies aimed at validating the predictive value of these biomarkers for long-term outcomes and early interventions in COVID-19 patients. Future research should focus on assessing the real-time applicability of these biomarkers in hospital settings.

A prospective design would provide valuable insights into the temporal changes in these biomarkers during hospitalization. A well-validated predictive model could pave the way for personalized care, leading to better outcomes, minimizing ICU overuse, and improving patient outcomes across diverse healthcare settings.

## 2. Materials and Methods

### Data Collection

***1.***  ***Study setting and population***

Our retrospective, observational study was conducted at the Prof. Dr. Matei Balș National Institute of Infectious Diseases, which is a major tertiary referral hospital in Bucharest, Romania, specializing in the management of infectious diseases. Our study included a total of 536 adult patients who were diagnosed with COVID-19 and admitted to Adult Ward 3 and Pediatric Ward XI. These wards were specifically designated for the treatment of COVID-19 patients during the study period, from 31 December 2020 to 8 October 2021.

The study was conducted at a time when the hospital was handling a high volume of COVID-19 cases, including both moderate and severe forms of the disease. Adult Ward 3 primarily accommodated adults with moderate to severe COVID-19 symptoms, including respiratory distress, and comorbidities such as hypertension, diabetes, and cardiovascular diseases. Due to the pandemic-related reorganization, Pediatric Ward XI also managed adult COVID-19 patients.

***2.***  ***Inclusion and Exclusion Criteria***

The inclusion criteria were as follows: age over 14 years, the ability to sign an informed consent, and blood tests collected within a maximum of 5 h after admission. All patients had a positive rapid diagnostic test or nasal or oropharyngeal RT-PCR. Native chest CT scans were performed on all participants at admission. Exclusion criteria included pregnant women, patients admitted to the intensive care unit per primam, and patients who did not undergo a native chest CT scan.

***3.***  ***Methods of Data Collection***

Data were collected during 31 December 2020–8 October 2021. All data were collected and organized in a structured database using Microsoft Excel. Data entry was performed by trained personnel. Our database was regularly checked for any missing or inconsistent data. Data analyses were conducted based on the data stored in this Excel database.

***4.***  ***Ethical Considerations***

The study protocol was approved by the ethics committee of INBIMB (protocol no. C0408/2020). All patients signed an informed consent, either personally or through an authorized representative, in the case of those aged between 14 and 18 years. All procedures were conducted in line with applicable guidelines and regulations, adhering to the principles outlined in the Declaration of Helsinki.

***5.***  ***Type of Data Collected***

Demographic data, including age and gender, as well as clinical parameters such as form of disease, signs and symptoms, co-pathologies, biochemical and hematological parameters such as inflammatory biomarkers (C-reactive protein, fibrinogen), complete blood count (CBC), cholestasis enzymes (gamma-glutamyl transferase, GGT) and cytolytic enzymes levels (alanine aminotransferase, ALT; aspartate aminotransferase, AST), complications and hematological indices, were collected.

***6.***  ***Statistical Analysis***

All statistical analyses were performed using IBM SPSS Statistics version 20 (IBM Corp., Armonk, NY, USA). Continuous variables were tested for normality using the Shapiro–Wilk test and were presented as medians with interquartile ranges (IQRs) for non-normally distributed data. Categorical variables were expressed as frequencies and percentages.

Comparisons between groups were conducted using the Mann–Whitney U test for non-normally distributed continuous data and independent samples t-test for normally distributed data. The chi-squared test or Fisher’s exact test was applied for categorical variables. Statistical significance was set at a *p*-value of <0.05 for all analyses.

Binary logistic regression was used to model the relationship between the dependent variable and one or more independent variables. Independent variables, which may include continuous or categorical predictors, were included in the model to assess their influence on the likelihood of the event occurring. The model’s fit was evaluated using tests such as the Hosmer–Lemeshow test. Statistical significance was determined using *p*-values, and odds ratios (ORs) with 95% confidence intervals (CIs) were calculated to interpret the strength and direction of the associations.

## 3. Results

We included 536 patients in the study, 41.6% women (*n* = 223) and 58.4% men (*n* = 313). The median age was 59 years (IQR: 47–70): 63 years (IQR: 52–72) in women and 54 years (IQR: 45–69) in men. The mean length of hospital stay was 10.94 days (SD: 5.86). Patients had multiple comorbidities, as listed above (Table 1).

A total of 386 patients (72%) had normal leukocyte values and 435 patients (81.2%) had normal neutrophil values. In contrast, only 82 patients (15.3%) had normal lymphocyte values (Table 2).

The median value for NLR was 5 (IQR: 3–8.39) and the median value for the PLR was 220.64 (IQR: 145.87–331.07); therefore, we considered the cut-off limit 5 for NLR and 220.64 for PLR. Moreover, 50.6% patients had a high NLR value (*n* = 271), and 50% patients had a high PLR value (*n* = 268). Half of the patients, 268 (50%), had both NLR and PLR over the cut off limit. No statistically significant differences were identified between the increased values of the two ratios and gender: *p* = 0.256, φ = 0.49.

Statistical correlations were identified between elevated NLR and PLR values and the development of complications during hospitalization (*p* = 0.04 and *p* = 0.00). Patients who presented with acute hypoxemic respiratory failure upon admission had elevated NLR and PLR levels (*p* = 0.00). Additionally, patients who required admission to the intensive care unit (ICU) exhibited significantly higher levels of both ratios (*p* = 0.04). Also, we obtained a statistically significant correlation between higher values of the two ratios and the possibility of developing a bacterial superinfection (*p* = 0.01, *p* = 0.04). However, no correlations were found between the values of these ratios and mortality (*p* = 0.46 and *p* = 0.32), nor the development of hepatic cytolysis (*p* = 0.79 and *p* = 0.87). We obtained statistical correlations between elevated levels of C-reactive (CRP) protein and complications that occurred during hospitalization (*p* = 0.02), the onset of acute hypoxemic respiratory failure (*p* = 0.00), the need for admission to the ICU (*p* = 0.03), and bacterial superinfection (*p* = 0.01). Moreover, we did not identify correlations between C-reactive protein levels and death (*p* = 0.16) or liver cytolysis occurring during hospitalization (*p* = 0.09). By comparison, such correlations were not observed with the independent values of leukocytes, lymphocytes, neutrophils, or CRP, except in relation to the need for initiating antibiotic therapy and CRP levels (Table 3 and Table 4).

A logistic regression analysis was performed to evaluate the ability of NLR, PLR, and CRP in predicting ICU admission. The overall model was statistically significant: Omnibus test: χ^2^ = 8.93, df = 3, *p* = 0.030, indicating that the combination of these biomarkers could significantly predict ICU admission. The model also demonstrated a good fit, using the Hosmer–Lemeshow test: χ^2^ = 6.218, df = 8, *p* = 0.62, suggesting that the model’s predicted probabilities align with the observed outcomes. The model’s discriminatory power, as measured by the area under the curve (AUC), was 0.710, indicating moderate predictive accuracy. Among the predictors, NLR, PLR, and CRP all showed significant contributions to the model, with each biomarker enhancing the ability to predict ICU admission.

Regarding complications, the logistic regression model was used to assess the predictive value of the two ratios and CRP in predicting the occurrence of complications. The overall model was statistically significant; χ^2^ = 9.675, df = 3, *p* = 0.022, suggesting that the combination of NLR, PLR, and CRP provides meaningful information for predicting complications. However, the individual contribution of NLR, PLR, and CRP was not statistically significant. Specifically, the B coefficient for the model was −0.082 (*p* = 0.354), and the odds ratio (Exp(B)) was 0.921, indicating no significant association between the predictors and the occurrence of complications.

Moreover, we constructed a logistic regression model to assess the predictive value of NLR, PLR, and CRP to the occurrence of bacterial superinfection. The overall model was statistically significant, χ^2^ = 9.675, df = 3, *p* = 0.022, meaning that the combination of NLR, PLR, and CRP provides important information for predicting bacterial superinfection, whose values were calculated to assess the discriminatory ability of each individual predictor: AUC = 0.557, PLR: AUC = 0.574, and CRP: AUC = 0.574. These AUC values suggest that each individual biomarker has moderate discriminatory power for predicting bacterial superinfection, but none of them show strong performance. In the logistic regression model, the B coefficient for the model was 0.941 (*p* = 0.000), and the odds ratio (Exp(B)) was 2.562, indicating that the combination of NLR, PLR, and CRP significantly increases the odds of bacterial superinfection. Furthermore, we obtained a good fit model: χ^2^ = 8.643, df = 8, *p* = 0.373.

The logistic regression analysis revealed a statistically significant negative association between C-reactive protein levels and the likelihood of ICU admission (B = −0.11, *p* = 0.003), with an odds ratio of 0.989, indicating a 1.1% decrease in the odds of ICU admission for each 1-unit increase in C-reactive protein. These data were confirmed by the Hosmer–Lemeshow test (chi-squared = 8.701, *p* = 0.368), suggesting that the model fits the data well. Despite the statistical significance, the small effect size indicates that C-reactive protein may have a modest impact on ICU admission decisions.

## 4. Discussion

NLR has been investigated as a simpler tool for diagnosing sepsis and can be calculated either using the absolute or relative counts of neutrophils and lymphocytes [17]. PLR, on the other hand, has been studied primarily for its role in evaluating the prognostic of cardiac diseases and malignant conditions [18].

The aim of this study was to explore the role of NLR and PLR in COVID-19, providing initial insights into their potential predictive value. Although retrospective in nature, our findings lay the foundation for prospective studies that can assess the real-time applicability and long-term impact of these biomarkers in clinical settings.

A recent study highlighted the importance of a hyperactive inflammatory response present in cancer patients, suggesting that a prognostic score, which includes NLR, PLR, C-reactive protein, and mGPS, could be used to assess the outcome of most cancers. Furthermore, the study emphasized that future research should focus on developing treatments that can moderate systemic inflammation in cancer patients, with the potential importance of these treatments being equal to, or even greater than, that of targeted cancer therapies themselves [19].

Additionally, another study demonstrated the significance of NLR during pregnancy, identifying a strong association between elevated NLR levels and the development of carotid wall thickening in otherwise healthy women [20].

NLR also shows clinical utility in urinary tract infections and respiratory viral infections, where clinicians in these studies were able to identify patients at risk of mortality. Moreover, in herpesvirus infections that progress to Ramsay Hunt syndrome, patients with higher NLR values were less likely to recover from their palsy [21].

These biomarkers provide valuable information on systemic inflammation, immune system activity, and the progression of various diseases as they can be derived from standard blood tests, and they represent cost-effective and widely accessible tools for both diagnostic and prognostic purposes [22]. Additionally, they align with the principles of personalized medicine by offering risk assessment, treatment options, and prognostic guidance tailored to individual patient profiles [23]. Unlike more complex molecular biomarkers, NLR and PLR can be easily calculated, ensuring their utility in diverse clinical settings worldwide.

The SARS-CoV-2 virus has the capacity of infecting the T lymphocytes by bonding to the angiotensin-converting enzyme 2 (ACE2) receptors and CD 147 spike proteins [24,25,26]. Severe forms of the disease lead to elevated levels of cytokines, which, along with lymphopenia, contribute to apoptosis and organ failure. Both CD4+ and CD8+ T cells are closely associated with the two ratios (NLR and PLR), as their levels increase in response to the rising values of these ratios [14,26,27,28].

Neutrophils play a central role in COVID-19 pathogenesis by driving immune processes like activation, degranulation, and cytokine production, which contribute to inflammation and lung injury. A key feature is that they are not involved in antibody-mediated virus elimination [29,30,31,32]. Neutrophil extracellular traps (NETs), while crucial for degrading pathogens, become dysregulated in severe COVID-19 and sepsis, contributing to the formation of the cytokine storm, which can then induce multiple organ dysfunction, including lung, renal, and neurological damage [32,33,34,35]. As COVID-19 progresses, platelets internalize circulating viral particles and activate TLR7, triggering complement component C3 release, which also promotes neutrophil extracellular trap formation (NETosis). Additionally, infection-induced tissue damage and inflammatory cytokines lead to thrombin generation and platelet aggregation, exacerbating microvascular thrombosis, especially in compromised endothelial tissues [36,37].

We identified statistical significance between elevated levels of the two ratios and the presence of hypoxemic acute respiratory failure, complications that developed during hospital stay and the need for antibiotic administration. Moreover, we found that ICU admission was correlated with elevated levels of these two ratios.

The logistic regression model demonstrated statistical significance in predicting the occurrence of bacterial superinfection. The model indicated that the combination of NLR, PLR, and CRP significantly increased the odds of bacterial superinfection. Specifically, for each unit increase in the combined values of these biomarkers, the odds of developing bacterial superinfection are more than two and a half times higher. We identified a moderate discriminatory power with an AUC of 0.710, with the ROC curve exhibiting a stepped, non-smooth appearance. This could indicate that we had few distinct predicted probabilities, potential model miscalibration, or data imbalances. The further calibration of the model’s predicted probabilities could improve the smoothness of the curve and enhance its overall performance.

Although the logistic regression model was statistically significant overall, the individual predictors did not significantly contribute to predicting the occurrence of complications. This suggests that, while these biomarkers collectively show promise in identifying risk factors for complications, they may not have a strong individual association with the outcome in this study.

The lack of significance might be attributed to a few factors, like the potential influence of confounding variables that were not included in the model, or the fact that the complications were grouped together despite their differences. Further studies are needed to investigate additional predictors or alternative approaches to identifying patients at risk for complications. Yet, the AUC values for each individual biomarker were relatively modest. These AUC values suggest that, while NLR, PLR, and CRP have some ability to discriminate between patients who develop bacterial superinfection and those who do not, their discriminatory power is limited and falls into the moderate range. The AUCs suggest that these biomarkers alone may not be strong enough for clinical decision-making without further context or additional predictive factors.

But they are a good fit model, indicating that the predicted probabilities align well with the observed outcomes, further supporting the reliability of the model. Even with the moderate AUC values, the combination of these biomarkers could still offer clinical utility in identifying patients at higher risk for bacterial superinfection, particularly in conjunction with other clinical indicators. Future studies with larger cohorts and additional biomarkers may enhance the predictive power of the model.

Although the logistic regression analysis demonstrated a statistically significant relationship between C-reactive protein levels and ICU admission, with a small decrease in the odds of ICU admission for each increase in C-reactive protein, the effect size was modest. This suggests that, while C-reactive protein may be statistically associated with ICU admission, its clinical impact in predicting ICU necessity is limited. The small magnitude of the association, coupled with the fact that other clinical factors likely play a more substantial role in ICU decision-making, indicates that C-reactive protein alone should not be relied upon as a primary criterion for ICU admission. Furthermore, the Hosmer–Lemeshow test confirmed that the model was well-fitted, but additional research is needed to explore the combined influence of C-reactive protein and other biomarkers, as well as clinical factors, to better understand their role in ICU triage and management. This interpretation highlights the statistical significance but emphasizes the modest clinical relevance and the need for further exploration of additional factors in ICU decision-making.

How effective are the two ratios compared to C-reactive protein, which is frequently used as a severity marker in clinical practice? Why would we choose NLR, PLR, or both combined instead of PCR? In addition to financial reasons, we identified that, although the level of C-reactive protein statistically correlates with the need for ICU admission, it cannot predict the potential progression of a patient towards ICU admission, like NLR and PLR do.

Moreover, although the high levels of CRP correlated statistically with the presence of acute respiratory failure, complications, and the need for initiation of antibiotic therapy, we also found that these results are influenced by the patient’s gender, in contrast to NLR and PLR, where no such correlation were found. No correlations were found regarding the influence of high levels of NLR and PLR and the presence of other chronic conditions such as aHTN, COPD, CKD, diabetes mellitus, or HIV.

Our study highlights the significant role of the NLR and PLR in predicting clinical outcomes in COVID-19 patients, specifically the presence of complications and acute respiratory failure. While CRP is a well-established marker of systemic inflammation, our findings emphasize that NLR and PLR offer additional advantages in clinical assessment. Both NLR and PLR demonstrated a strong correlation with acute respiratory failure and complications, like CRP, but with key differences in their potential applications.

The primary advantage of using NLR and PLR is their accessibility and cost-effectiveness. Unlike CRP, which may require more extensive testing, both NLR and PLR can be easily calculated from routine blood tests, making them highly practical for early risk stratification and decision-making in clinical settings. Moreover, our results indicate that these ratios may offer more precise insights into the inflammatory response, which is crucial in determining the severity of disease progression in COVID-19 patients.

While CRP remains a valuable marker for inflammation, NLR and PLR provide additional layers of prognostic information, especially in predicting adverse outcomes such as ICU admission and respiratory failure. Therefore, NLR and PLR should be considered as complementary tools to CRP, for more precise and prompt clinical interventions.

## 5. Conclusions

Our study highlights the potential value of combining NLR, PLR, and CRP in predicting adverse outcomes in hospitalized patients. We found statistically significant associations between elevated levels of these ratios and critical clinical events, including hypoxemic acute respiratory failure, the development of complications during hospitalization, and the need for antibiotic administration. Notably, ICU admission was correlated with elevated levels of these biomarkers, although no significant association was observed with mortality.

Interestingly, although the biomarkers collectively showed predictive potential, individual predictors did not significantly contribute to forecasting complications in isolation. This finding highlights the importance of considering these biomarkers in combination rather than individually when assessing patient risk.

Overall, our findings suggest that NLR and PLR hold promise as a combined tool for identifying patients at risk for bacterial superinfection and other complications. The further refinement of the predictive model and validation in larger, more diverse populations are necessary to confirm its clinical utility and optimize its application in practice.

In the future, NLR and PLR could serve as practical tools for the early identification of high-risk COVID-19 patients and for monitoring disease progression. Prospective studies could evaluate how these biomarkers change over the course of infection, allowing clinicians to better predict which patients are at risk of severe complications. By tracking NLR and PLR levels, future research could provide insights into the timing and adjustment of treatments, helping to determine when interventions such as corticosteroids, anticoagulants, or immunomodulatory drugs should be initiated.

Furthermore, clinical trials could explore whether reducing neutrophil or platelet activation through targeted therapies improves patient outcomes, making NLR and PLR valuable markers for assessing treatment effectiveness. As simple, cost-effective biomarkers, NLR and PLR could be integrated into routine clinical practice to enhance decision-making and personalize care for COVID-19 patients. In the long term, these findings could contribute to broader research on inflammation-related diseases, improving the management of both infectious and inflammatory conditions.

In conclusion, while our study lays the groundwork for utilizing NLR and PLR as predictive biomarkers in COVID-19, prospective studies are crucial to validate these findings and refine their role in clinical decision-making. Such studies could ultimately contribute to the development of a multi-biomarker scoring system that helps healthcare providers make more accurate and timely decisions, improving patient outcomes and optimizing the use of resources in critical care settings.

## Figures and Tables

**Table 1:** Co-existing conditions in our study population.

	Total *n* (%)	Men *n* (%)	Women *n* (%)
aHTN *	270 (50.4)	141 (45)	129 (57.8)
T2DM **	74 (13.8)	39 (12.5)	35 (15.7)
T1DM ***	29 (5.4)	20 (6.4)	9 (4)
COPD ****	13 (2.4)	9 (2.9)	4 (1.8)
CKD *****	28 (5.2)	22 (7)	6 (2.7)
Dialysis	5 (0.9)	3 (1)	2 (0.9)
Hematological diseases	12 (2.2)	6 (1.9)	6 (2.7)
Active neoplasia	28 (5.2)	14 (4.5)	14 (6.3)
History of a neoplasia	34 (6.3)	8 (2.6)	26 (11.7)
Chronic liver disease	45 (8.4)	30 (9.6)	15 (6.7)
HIV	1 (0.2)	1 (0.3)	0
Autoimmune diseases	25 (4.7)	10 (3.2)	15 (6.7)
Immunosuppression	8 (1.5)	6 (1.9)	2 (0.9)
Other chronic conditions	363 (67.7)	198 (63.3)	165 (74)

Note: data are expressed as n (%) Abbreviations: * aHTN, arterial hypertension; ** T2DM, non-insulin-dependent (type II) diabetes mellitus; *** T1DM, insulin-dependent (type I) diabetes mellitus; **** COPD, chronic obstructive pulmonary disease; ***** CKD, chronic kidney disease.

**Table 2:** Baseline laboratory findings upon admission.

Number of Cases (%)		
WBC 4.0–10.0 × 10^3^/µL	<4.0 × 10^3^/µL	79 (14.7)
	4.0–10.0 × 103/µL	386 (72)
	>10.0 × 10^3^/µL	70 (13.1)
Neu 1.8–8.0 × 10^3^/µL	<1.8 × 10^3^/µL	20 (3.7)
	1.8–8.0 × 10^3^/µL	435 (81.2)
	>8.0 × 10^3^/µL	81 (15.1)
Lym 1.5–4.0 × 10^3^/µL	<1.5 × 10^3^/µL	449 (83.8)
	1.5–4.0 × 10^3^/µL	82 (15.3)
	>4.0 × 10^3^/µL	5 (0.9)
Plt 200–400 × 10^3^/µL	<200.00 × 10^3^/µL	256 (47.8)
	200–400 × 10^3^/µL	252 (47)
	>400 × 10^3^/µL	28 (5.2)

Note: data are expressed as n (%) Abbreviations: WBC, white blood cell; Neu, neutrophil; Lym, lymphocyte; Plt, platelet.

**Table 3:** Statistical analysis of ratios and their association with clinical outcomes: high vs. normal values. Abbreviations: ICU, intensive care unit; ARF, acute respiratory failure; CRP, C-reactive protein.

Ratios	All Cases N (%)	High Values	Normal Values	Statistical Significance
NLR				
Deceased	4 (0.7)	2	2	*p* = 0.46
ICU	8 (1.5)	7	1	*p* = 0.04
ARF	391 (64)	283	122	*p* = 0.00
Complications	279 (52.1)	220	59	*p* = 0.04
Bacterial superinfection	148 (27.6)	119	29	*p* = 0.01
CRP	485 (90.5)	375	110	*p* = 0.003
Fibrinogen	431 (80.4)	339	92	*p* = 0.001
Hepatic cytolysis	298 (55.6%)	226	72	*p* = 0.79
Cholestasis	312 (58.2)	244	68	*p* = 0.093
PLR				
Deceased	4 (0.7)	2	2	*p* = 0.32
ICU	8 (1.5)	7	1	*p* = 0.04
ARF	391 (64)	271	72	*p* = 0.00
Complications	279 (52.1)	219	60	*p* = 0.00
Bacterial superinfection	148 (27.6)	117	31	*p* = 0.04
CRP	485 (90.5)	364	121	*p* = 0.001
Fibrinogen	431 (80.4)	330	101	*p* = 0.000
Hepatic cytolysis	298 (55.6%)	225	73	*p* = 0.87
Cholestasis	312 (58.2)	242	70	*p* = 0.005

**Table 4:** Statistic correlations found between blood parameters and clinical outcomes. Abbreviations: CRP, C-reactive protein; NLR, neutrophil-to-lymphocyte ratio; PLR, platelet-to-lymphocyte ratio; ARF, acute respiratory failure; ICU, intensive care unit.

	Gender	ARF	Complications	Bacterial Superinfection	ICU	Death
Leu	*p* = 0.00	*p* = 0.19	*p* = 0.61	*p* = 0.16	*p* = 0.56	*p* = 0.88
Lym	*p* = 0.98	*p* = 0.00	*p* = 0.03	*p* = 0.07	*p* = 0.02	*p* = 0.67
Neu	*p* = 0.00	*p* = 0.00	*p* = 0.27	*p* = 0.19	*p* = 0.27	*p* = 0.60
Plt	*p* = 0.04	*p* = 0.06	*p* = 0.13	*p* = 0.34	*p* = 0.84	*p* = 0.24
CRP	*p* = 0.00	*p* = 0.00	*p* = 0.26	*p* = 0.01	*p* = 0.03	*p* = 0.16
NLR	*p* = 0.08	*p* = 0.00	*p* = 0.04	*p* = 0.01	*p* = 0.04	*p* = 0.46
PLR	*p* = 0.44	*p* = 0.00	*p* = 0.00	*p* = 0.04	*p* = 0.04	*p* = 0.32

## Data Availability

The datasets presented in this article are not readily available because they are part of an ongoing PhD thesis. Requests to access the datasets should be directed to the main author.
